# The effectiveness and safety of platinum-based pemetrexed and platinum-based gemcitabine treatment in patients with malignant pleural mesothelioma

**DOI:** 10.1186/s12885-015-1519-z

**Published:** 2015-07-09

**Authors:** Guntulu Ak, Selma Metintas, Muhittin Akarsu, Muzaffer Metintas

**Affiliations:** 1 Lung and Pleural Cancers Research and Clinical Center, Eskisehir Osmangazi University, Eskisehir, Turkey; 2 Department of Chest Diseases, Eskisehir Osmangazi University, Medical Faculty, Eskisehir, Turkey; 3 Department of Public Health, Eskisehir Osmangazi University, Medical Faculty, Eskisehir, Turkey

## Abstract

**Background:**

We aimed to evaluate the efficiency and safety of cis/carboplatin plus gemcitabine, which was previously used for mesothelioma but with no recorded proof of its efficiency, compared with cis/carboplatin plus pemetrexed, which is known to be effective in mesothelioma, in comparable historical groups of malignant pleural mesothelioma.

**Methods:**

One hundred and sixteen patients received cis/carboplatin plus pemetrexed (group 1), while 30 patients received cis/carboplatin plus gemcitabine (group 2) between June 1999 and June 2012. The two groups were compared in terms of median survival and adverse events to chemotherapy.

**Results:**

The mean ages of groups 1 and 2 were 60.7 and 60.8 years, respectively. Most of the patients (78.1 %) had epithelial type tumors, and 47 % of the patients had stage IV disease. There was no difference between the two groups in terms of age, gender, asbestos exposure, histology, stage, Karnofsky performance status, presence of pleurodesis, prophylactic radiotherapy, second–line chemotherapy and median hemoglobin and serum albumin levels. The median survival time from diagnosis to death or the last day of follow up with a 95 % confidence interval was 12 ± 0.95 months (95 % CI: 10.15–13.85) for group 1 and 11.0 ± 1.09 months (95 % CI: 8.85–13.15) for group 2 (Log-Rank: 0.142; *p* = 0.706). The median survival time from treatment to death or the last day of follow-up with a 95 % confidence interval was 11.0 ± 0.99 months (95 % CI: 9.06–12.94) for group 1 and 11.0 ± 1.52 months (95 % CI: 8.02–13.97) for group 2 (Log-Rank: 0.584; *p* = 0.445). The stage and Karnofsky performance status were found to be significant variables on median survival time by univariate analysis. After adjusting for the stage and Karnofsky performance status, the chemotherapy schema was not impressive on median survival time (OR: 0.837; 95 % CI: 0.548–1.277; *p* = 0.409). The progression free survival was 7.0 ± 0.61 months for group I and 6.0 ± 1.56 months for group II (Log-Rank: 0.522; *p* = 0.470). The treatment was generally well tolerated, and the side effects were similar in both groups.

**Conclusions:**

The study indicates that platinum-based gemcitabine is effective and a safe schema in malignant pleural mesothelioma. Further research should include large randomized phase III trials comparing these agents.

## Background

Malignant pleural mesothelioma (MPM) is an aggressive tumor and remains a significant worldwide health problem because of its poor prognosis and increasing incidence [1]. Most of the patients have advanced disease at diagnosis and are not eligible for multimodality treatment. Platinum–based chemotherapy is the most effective systemic therapy in patients with advanced stage disease. Currently, following a randomized phase III study, the combination of cisplatin and pemetrexed is widely used for the systemic treatment of advanced disease [2]. Another randomized phase III study of cisplatin and raltitrexed in MPM showed similar results [3]. However, there is currently no universally accepted standard chemotherapeutic regimen for MPM.

The combination of cisplatin and gemcitabine was widely used for MPM throughout the world before the combination of antifolates and platinum compounds. Some studies reported that the combination of platinum and gemcitabine is also a reasonable first–line option for the systemic therapy of MPM because of its acceptable toxicity profile, good response and survival rates, and symptom-relieving effects [4–11]. Although gemcitabine in combination with cisplatin or carboplatin shows definite activity in MPM, given the lack of phase III evidence, the use of gemcitabine as a first–line therapy is not supported. Gemcitabine in combination with platinum or alone is being used in the clinic as a second–line regimen for MPM.

There are few studies compared the two regimens. They showed that platinum – based doublets might represent similar therapeutic activity in MPM [12–14]. Pemetrexed is an expensive agent, and when it is used, folic acid and vitamin B12 supplementation are required to reduce the toxicity. In our study, we aimed to evaluate the efficiency and safety of cis/carboplatin plus gemcitabine compared with cis/carboplatin plus pemetrexed, in comparable historical groups of malignant pleural mesothelioma.

## Methods

### Patients

Between June 1999 and June 2012, a total of 343 patients were histologically diagnosed with MPM at the Chest Disease Department of Eskisehir Osmangazi University Hospital in Turkey. After diagnosis, the best supportive care, chemotherapy, surgery, radiotherapy or combination therapies were given to the patients in the Pulmonary Oncology Unit of the Chest Disease Department. The study was approved by the Ethical Committee of Eskisehir Osmangazi University (number: 08.03.2000/242), and all of the patients provided written informed consent. A total of 146 patients, who had platinum compounds in combination with gemcitabine or pemetrexed, were enrolled in this study. The study inclusion criteria were as follows: no prior therapy except local radiotherapy to the entry site; measurable or evaluable disease; KPS of 70–100; age greater than 18 years; and adequate bone marrow (leukocytes ≥ 3000 / μL, granulocytes ≥ 1500 / μL, hemoglobin ≥ 10 g / dL, and platelets ≥ 100,000 μL), renal (creatinine ≤ 1.5 mg / dL or creatinine clearance ≥ 60 mL / min), hepatic (total bilirubin within normal institutional limits and AST and ALT ≤ 2.5 X upper limit of normal), and coagulation (prothrombin time international normalized ratio ≤ 1.5) functions were required. All of the patients provided written informed consent before chemotherapy according to our institutional guidelines. One hundred and ninety-seven patients were excluded from the study: 81 patients had only the best supportive care; 62 patients had surgical treatment; 22 patients received different chemotherapeutic regimens as first–line treatment; 2 patients died due to reasons unrelated to MPM or chemotherapy (pulmonary embolism and pneumonia); 20 patients were not be able to evaluate for a chemotherapy response; and 10 patients were not followed.

Clinical data, including age, gender, history of asbestos exposure, histology, stage, Karnofsky Performance Status (KPS), treatment history, side effects, response to chemotherapy, pleurodesis, prophylactic radiotherapy, second–line chemotherapy, baseline hemoglobin, baseline serum albumin and survival characteristics were collected for all the patients. All of the patients were staged according to the International Mesothelioma Interest Group (IMIG) staging system [15]. The tumor response was evaluated using a modified Response Evaluation Criteria in Solid Tumors (RECIST) [16].

### Treatment

The patients were classified into two groups according to their chemotherapy regimen: 116 patients received cis/carboplatin and pemetrexed (group 1), and 30 patients received cis/carboplatin and gemcitabine (group 2). The two groups were compared in terms of epidemiological, clinical and survival characteristics. The side effects of chemotherapy were also recorded.

In the pulmonary oncology unit of our clinic, cis/carboplatin and gemcitabine regimen was used as a first – line chemotherapy regimen until October 2005. Cis/carboplatin and pemetrexed has been used since October 2005. Gemcitabine 1250 mg/m^2^ was given intravenously on days 1 and 8 of a 21-day cycle, followed by intravenous cisplatin 75 mg/m^2^ or carboplatin 300 mg/m^2^ on day 1. Pemetrexed 500 mg/m^2^ was given intravenously on day one, followed by cisplatin 80 mg/m^2^ or carboplatin 300 mg/m^2^, intravenously on day 1, which was repeated every 21 days. Before the administration of pemetrexed, folic acid, vitamin B12, and dexamethasone supplementation were provided. Antiemetic therapy with 5-hydroxytryptamine antagonists and dexamethasone was given intravenously on day 1 and, thereafter, orally for 3 to 5 days. Chemotherapy was given for 4 to 6 cycles or until disease progression, unacceptable adverse events, or patient unwillingness to chemotherapy. Additionally, the use of any second-line chemotherapy was recorded. Adverse events were graded according to the World Health Organization (WHO) criteria and were assessed before chemotherapy [17]. All the side effects of chemotherapy were recorded. Dose adjustments for adverse events were based on a stepwise reduction schedule.

A history and physical examination, complete blood count and differential, chemistry panel, electrocardiogram, chest radiograph, and chest and abdominal computed tomography (CT) scans were performed at baseline. Bone scans and brain magnetic resonance imaging were performed only if clinically indicated. Thereafter, the history and physical examination were performed every 21 days. The complete blood count and differential and chemistry panel were performed weekly. The tumor response to chemotherapy was evaluated by computed tomography (CT) scans, obtained every two or three cycles of chemotherapy. Thereafter, CT scans were performed at 3 monthly intervals until disease progression.

### Statistical analysis

Data were collected, analyzed, and evaluated in the Lung and Pleural Cancers Research and Clinical Center of Eskisehir Osmangazi University. All of the analyses were performed using a statistical software (SPSS, version 11.5). The characteristics of the patients, according to their chemotherapy schedule, were compared using a *t*-test for continuous variables and Pearson *X*^2^ test or Fisher’s Exact test for frequency. The median hemoglobin and serum albumin levels were compared using Mann–Whitney *U* test. The median survival times and progression – free survival with 95 % confidence intervals (CI) were estimated for each group. The survival curves were generated using the Kaplan – Meier method. All of the patients were followed until death or for a minimum period of 1 year. The median survival times were compared between the chemotherapy groups using both an unadjusted and a stratified (by stage, histology, KPS) log-rank test. The Cox proportional hazards regression models were fit to assess the effect of treatment, stage, histology, KPS, and other potential prognostic factors of survival. The interactions between treatments and stratification factors were explored using the Cox model. The significance level was considered to be 5 % (*p* < 0.05), and the approach used was bilateral.

## Results

One hundred and sixteen patients received cis/carboplatin plus pemetrexed (group 1) between October 2005 and June 2012, and 30 patients received cis/carboplatin plus gemcitabine (group 2) between June 1999 and September 2005. The mean ages of groups 1 and 2 were 60.7 and 60.8 years, respectively. The female ratios of groups 1 and 2 were 50 % and 53 %, respectively. Most of the patients (98 %) were exposed to asbestos for a major part of their life. Most of the patients (78.1 %) had epithelial type tumors, and approximately half of them had stage IV disease (47 %).

There was no difference between the two groups in terms of age, sex, asbestos exposure, KPS, histological cell type, stage, presence of pleurodesis, prophylactic radiotherapy, second–line chemotherapy and baseline hemoglobin and serum albumin levels (Table 1). These results showed that they were comparable groups.

**Table 1:** Patient demographics and clinical characteristics

Demographics or clinical characteristics	Cis/carboplatin and pemetrexed (n = 116)	Cis/carboplatin and gemcitabine (n = 30)	*p* value
Mean age, years	60.7 ± 10.9	60.8 ± 9.9	0.945
Male: Female	58 : 58	14 : 16	0.745
Asbestos exposure, n (%)	113 (97.4)	29 (96.7)	0.827
Mean Karnofsky Performance Status	85.7 ± 9.9	80.3 ± 8.7	0.062
Histology, n (%)			
Epithelial	92 (79.3)	22 (73.3)	1.00
Sarcomatous	9 (7.8)	2 (6.7)	
Mixed	12 (10.3)	5 (16.7)	
Unidentified	3 (2.6)	1 (3.3)	
Stage, n (%)			
I	6 (5.2)	2 (6.7)	0.662
II	12 (10.4)	2 (6.7)	
III	41 (35.7)	14 (46.7)	
IV	56 (48.7)	12 (40.0)	
Pleurodesis, n (%)	41 (35.3)	10 (33.3)	0.837
Prophylactic radiotherapy, n (%)	22 (19.0)	2 (6.7)	0.083
Second–line chemotherapy, n (%)	36 (31.0)	8 (26.7)	0.642
Median baseline hemoglobin, g / dL	12.5	12.3	0.679
Median baseline serum albumin, g / dL	3.8	3.6	0.336

There was no significant difference between the objective response rates to chemotherapy in the two treatment groups: 32.7 % for group I, and 36.7 % for group II (*p* = 0.700). The complete response rates were 3.4 % vs. 6.7 %, while the partial response rates were 29.3 % vs. 30.0 % in groups I and II, respectively. Stable disease occurred in 39.7 % and 30 % of the patients in groups I and II, respectively. The one-year survival rate for group I was 58.6 % and 56.6 % for group II; the 2–year survival rate for group I was 20.7 % and 13.3 % for group II; the 3 – year survival rate was 9.5 % for group I and 10 % for group II.

**Fig. 1:**
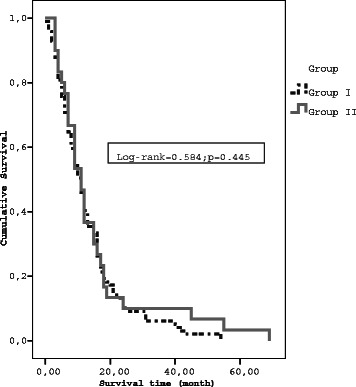
Median survival by treatment groups

The median survival time from diagnosis to death or the last day of follow-up with a 95 % confidence interval was 12.0 ± 0.95 months (95 % CI: 10.15–13.85) for group 1 and 11.0 ± 1.09 months (95 % CI: 8.85–13.15) for group 2. The median survival time of the two groups was not different (Log-Rank: 0.142; *p* = 0.706). The median survival time from treatment to death or the last day of follow-up with a 95 % confidence interval was 11.0 ± 0.99 months (95 % CI: 9.06–12.94) for group 1 and 11.0 ± 1.52 months (95 % CI: 8.02–13.97) for group 2 (Log-Rank: 0.584; *p* = 0.445) (Fig. 1).

Stage and KPS were found as significant variables of median survival time by univariate analysis in the two groups (*p* = 0.002 and *p* = 0.045, respectively). After adjusting for stage and KPS, the chemotherapy regimen was not impressive on median survival time (OR: 0.837; 95 % CI: 0.548–1.277; *p* = 0.409).

**Fig. 2:**
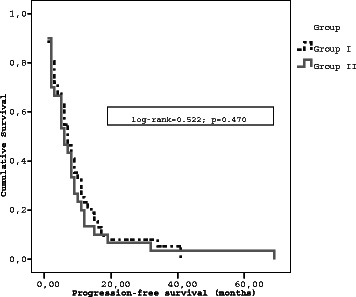
Progression – free survival by treatment groups

The progression free survival was 7.0 ± 0.61 months (95 % CI: 5.079–8.20) for group I and 6.0 ± 1.56 months (95 % CI: 2.94–9.06) for group II (Log-Rank: 0.522; *p* = 0.470) (Fig. 2).

The treatment was generally well tolerated, and the side effects were similar in both the groups (Table 2).

**Table 2:** Grade III and IV toxicities according to treatment groups

Toxicity	Group I	Group II	P*
	n (%)	n (%)	
Neutropenia	19 (16.3)	3 (10.0)	0.568
Anemia	4 (3.4)	1 (3.3)	1.000
Thrombocytopenia	6 (5.1)	4 (13.3)	0.123
Nause / vomiting	8 (6.9)	5 (16.6)	0.142
Nephrotoxicity	5 (4.3)	-	0.584
Febrile neutropenia	2 (1.7)	2 (6.6)	0.187

Group I: cis/carboplatin + pemetrexed; Group II: cis/carboplatin + gemcitabine; a: Only the worst WHO grade was reported for each patient

*: Fisher’s Exact Test

## Discussion

There is currently no widely accepted therapy for MPM. The necessity for more effective treatments for patients with mesothelioma is obvious. A number of novel agents have been assessed to date. Several classes of drugs are being explored, including those that affect DNA transcription, cell-cycle progression, angiogenesis, and immune tolerance. Several ongoing or recently completed phase II and III trials using novel agents such as vorinostat, everolimus, CBP501, MORAb-009, NGR-hTNF, WT1 vaccine, bevacizumab, cediranib, and thalidomide have been conducted [18]. Although some of those targeted therapies have seemed to be promising, none of these agents have been implemented in daily practice. Therefore, most efforts are directed towards improving standard first-line therapy with antifolates and platinum compounds, or developing effective second-line treatments.

Platinum–based chemotherapy seems to be the most effective systemic therapy in patients with advanced stage disease. Platinum based gemcitabine was widely used in the late 1990s and 2000s as a part of phase studies in MPM because of the synergy between gemcitabine and platinum compounds [4–11]. In those studies, 9.6 to 13 months of overall survival time and 12 % to 48 % of the response rates were reported [4–11]. Variable activity has been observed with gemcitabine and platinum compounds in those studies because of small sample size, heterogeneity in prognostic factors, and different response measurements. Additionally, the symptom control was better than the control arm in some of these studies [4, 6, 7, 10]. Although the results have been variable, the response rate and survival data of the studies have been encouraging in advanced stage mesothelioma. While some physicians recommend only supportive care for mesothelioma, platin-based gemcitabine has become a widely used regimen in front-line chemotherapy for mesothelioma in some clinics. We used platin-based gemcitabine before pemetrexed, between June 1999 and September 2005, for mesothelioma in our clinic in Turkey.

Two large randomized phase III trials with combinations of antifolate and cisplatin have demonstrated a survival advantage with this combination compared with single agent cisplatin [2, 3]. In the trial comparing cisplatin plus pemetrexed to cisplatin alone, the response rate with the combination was 41 % in comparison to 17 % with cisplatin [2]. The median survival time was 12.1 months with the combination regimen. In the other phase III study with raltitrexed, the response rate was 23 %, with a median survival time of 11.4 months [3]. Following the study of Vogelzang et al., platin-based pemetrexed was approved by the FDA as a first-line treatment in mesothelioma. Thereafter, within several years, platinum and antifolate, especially pemetrexed, has been established as a standard of care in front-line chemotherapy for mesothelioma worldwide. Physicians have some concern about these studies. Neither cisplatin-based pemetrexed nor raltitrexed were compared with another doublet. Both studies used only cisplatin in the control arm. Interestingly, there is not much interest in raltitrexed. First, they are expensive agents, especially pemetrexed. When increasing incidence of mesothelioma is taken into account, pemetrexed is not cost effective for most of the patients especially in developing countries. Additionally, supplementation of folic acid and vitamin B12 is needed to reduce the toxicity of pemetrexed.

Although, following the study of Vogelzang et al. in 2003, cisplatin-based pemetrexed has been established as first-line chemotherapy for mesothelioma in most of the world, gemcitabine and cisplatin is still accepted as an effective treatment regimen in phase II studies [19, 20].

There is not sufficient number of study compared platinum based doublets such as pemetrexed, raltitrexed, gemcitabine and vinorelbine with one another. We hypothesize that those doublets that are platin plus pemetrexed and platin plus gemcitabine may be comparable in terms of efficacy and safety, based on previous phase II studies of gemcitabine. The good response rate and median survival time observed in our study suggest that the combination of platin plus gemcitabine may have similar effects compared to pemetrexed plus cisplatin.

Lee et al. reviewed platinum analogs with either gemcitabine or pemetrexed in 81 mesothelioma patients, retrospectively [12]. They reported similar overall survival and 1– and 2–year survival rates with both regimens. However, in their study, the survival was better in the group that was able to receive second–line systemic therapy. Another study comparing gemcitabine plus cisplatin and pemetrexed plus carboplatin came from Cairo by Habib et al [13]. They set up a randomized phase II study that included 40 patients with mesothelioma. In their study, the response was superior in the pemetrexed group (78.9 % vs. 47.6 %). However, cumulative survival at 1.5 years was similar in the two groups. Additionally, patients included in the pemetrexed group were younger than the patients in the gemcitabine group (49 years old vs. 62 years old; *p* < 0.001). Age is a significant prognostic factor in mesothelioma and may be responsible for the good response to pemetrexed. Another small study, including total 30 patients, did not show a significant difference in terms of response rate and overall survival time between the two groups [14].

In the current study, the regimens were well tolerated with no toxic deaths. The frequencies and severity of toxicities experienced with platin plus gemcitabine or pemetrexed regimens appear to be comparable to those found in the literature.

## Conclusions

In conclusion, the study indicates that platinum-based gemcitabine is effective and a safe schema in malignant pleural mesothelioma. To provide a more cost-effective treatment approach for advanced MPM, further research should include randomized controlled phase III trials comparing platinum doublets plus antifolate and platinum doublets plus gemcitabine or vinorelbine.

## References

[1] Ray M, Kindler HL (2009). Malignant pleural mesothelioma: an update on biomarkers and treatment. Chest.

[2] Vogelzang NJ, Rusthoven JJ, Symanowski J, Denham C, Kaukel E, Ruffie P, Gatzemeier U, Boyer M, Emri S, Manegold C, Niyikiza C, Paoletti P (2003). Phase III study of pemetrexed in combination with cisplatin versus cisplatin alone in patients with malignant pleural mesothelioma. J Clin Oncol.

[3] van Meerbeeck JP, Gaafar R, Manegold C, van Klaveren RJ, van Marck EA, Vincent M, Legrand C, Bottomley A, Debruyne C, Giaccone G (2005). Randomized phase III study of cisplatin with or without raltitrexed in patients with malignant pleural mesothelioma: an intergroup study of the European Organization for Research and Treatment of Cancer Lung Cancer Group and the National Cancer Institute of Canada. J Clin Oncol.

[4] Byrne MJ, Davidson JA, Musk AW, Dewar J, van Hazel G, Buck M, de Klerk NH, Robinson BW (1999). Cisplatin and gemcitabine treatment for malignant mesothelioma: a phase II study. J Clin Oncol.

[5] Van Haarst JMW, Baas P, Manegold C, Schouwink JH, Burgers JA, de Bruin HG, Mooi WJ, van Klaveren RJ, de Jonge MJ, van Meerbeeck JP (2002). Multicentre phase II study of gemcitabine and cisplatin in malignant pleural mesothelioma. Br J Cancer.

[6] Nowak AK, Byrne MJ, Williamson R, Ryan G, Segal A, Fielding D, Mitchell P, Musk AW, Robinson BW (2002). Multicentre phase II study of cisplatin and gemcitabine for malignant mesothelioma. Br J Cancer.

[7] Favaretto AG, Aversa SML, Paccagnella A, Manzini VP, Palmisano V, Oniga F, Stefani M, Rea F, Bortolotti L, Loreggian L, Monfardini S (2003). Gemcitabine combined with carboplatin in patients with malignant pleural mesothelioma. Cancer.

[8] Schutte W, Blankenburg T, Lauerwald K, Schreiber J, Bork I, Wollscgkaeger B, Treutler D, Schneider CP, Bonner R (2003). A multicenter phase II study of gemcitabine and oxaliplatin for malignant pleural mesothelioma. Clin Lung Cancer.

[9] Castagneto B, Zai S, Dongiovanni D, Muzio A, Bretti S, Numico G, Botta M (2005). Cisplatin and gemcitabine in malignant pleural mesothelioma: a phase II study. Am J Clin Oncol.

[10] Utkan G, Buyukcelik A, Yalcin B, Akbulut H, Demirkazik A, Dincol D, Onur H, Goren D, Mousa U, Senler FC, Icli F (2006). Divided dose of cisplatin combined with gemcitabine in malignant mesothelioma. Lung Cancer.

[11] Kalmadi SR, Rankin C, Kraut MJ, Jacobs AD, Petrylak DP, Adelstein DJ, Keohan ML, Taub RN, Borden EC (2008). Gemcitabine and cisplatin in unresectable malignant mesothelioma of the pleura: a phase II study of the Southwest Oncology Group (SWOG 9810). Lung Cancer.

[12] Lee CW, Murray N, Anderson H, Rao SC, Bishop W (2009). Outcomes with first-line platinum-based combination chemotherapy for malignant pleural mesothelioma: a review of practice in British Columbia. Lung Cancer.

[13] Habib EE, Fahmy ES (2013). Chemotherapy management of malignant pleural mesothelioma: a phase II study comparing two popular chemotherapy regimens. Clin Transl Oncol.

[14] Shukuya T, Takahashi T, Imai H, Tokito T, Ono A, Akamatsu H, Taira T, Kenmotsu H, Naito T, Murakami H, Endo M, Yamamoto N (2014). Comparison of cisplatin plus pemetrexed and cisplatin plus gemcitabine for the treatment of malignant pleural mesothelioma in Japanese patients. Respir Investig.

[15] Rusch VW (1995). A proposed new international TNM staging system for malignant pleural mesothelioma. From the International Mesothelioma Interest Group. Chest.

[16] Byrne MJ, Nowak AK (2004). Modified RECIST criteria for assessment of response in malignant pleural mesothelioma. Ann Oncol.

[17] Miller AB, Hoogstraten B, Staquet M, Winkler A (1981). Reporting results of cancer treatment. Cancer.

[18] Zauder MG, Krug LM (2012). Novel therapies in phase II and III trials for malignant pleural mesothelioma. J Natl Compr Canc Netw.

[19] Kindler HL, Karrison TG, Gandara DR, Lu C, Krug LM, Stevenson JP, Janne PA, Quinn DI, Koczywas MN, Brahmer JR, Albain KS, Taber DA, Armato SG, Vogelzang NJ, Chen HX, Stadler WM, Vokes EE (2012). Multicenter, double-blind, placebo-controlled, randomized phase II trial of gemcitabine/cisplatin plus bevacizumab or placebo in patients with malignant mesothelioma. J Clin Oncol.

[20] Arrieta O, Lopez-Macias D, Mendoza-Garcia VO, Bacon-Foseca L, Munoz-Montano W, Macedo-Perez EO, Muniz-Hernandez S, Blake-Cerda M, Corona-Cruz JF (2014). A phase II trial of prolonged, continuous infusion of low-dose gemcitabine plus cisplatin in patients with advanced malignant pleural mesothelioma. Cancer Chemother Pharmacol.

